# Apolar Annonaceous Acetogenins from the Fruit Pulp of *Annona muricata*

**DOI:** 10.3390/molecules14114387

**Published:** 2009-11-02

**Authors:** Alice Melot, Djibril Fall, Christophe Gleye, Pierre Champy

**Affiliations:** 1 Laboratoire de Pharmacognosie, CNRS UMR 8076 BioCIS, Faculté de Pharmacie Paris-Sud 11, Rue J. B. Clément, 92296 Châtenay-Malabry, France; 2 Laboratoire de Chimie Thérapeutique, Faculté de Pharmacie, Université Cheikh Anta Diop, Dakar, Senegal; E-Mail: djibril_fall@yahoo.fr (D.F.)

**Keywords:** *Annona muricata* L., annonaceae, annonaceous acetogenin

## Abstract

A methylene chloride extract of the pulp of *Annona muricata* L. was fractionated in search for scarcely functionalized Annonaceous acetogenins (type E). Previously known C-35 and C-37 mono-epoxy unsaturated compounds, epomuricenins-A and -B (**1+2**) and epomusenins-A and -B (**3+4**), were obtained. Two new mono-epoxy saturated C-35 representatives, epomurinins-A and -B (**5+6**) were also isolated.

## Introduction

*Annona muricata* L., a fruit tree growing in tropical and sub-tropical areas, is one of the main cultivated species of the genera, whose fruit is consumed either raw of after processing. This foodstuff is suspected of being implicated in the occurrence of atypical parkinsonian syndromes in the French West Indies [1, *and see* 2 in this issue]. The presence of Annonaceous acetogenins (ACGs) [3,4,5] of various polarities was assessed in the pulp, from which annonacin, the main neurotoxic candidate in the species, was isolated [6]. In the course of this chemical investigation, existence of precursors of THF-bearing ACGs (*i.e.*, ACGs of type E) was suspected, but could not be ascertained, from observations with Kedde reagent-visualized thin layer chromatography (see Experimental section) or RP-HPLC-UV. MALDI-TOF MS (Matrix-Assisted Laser Desorption Ionization Time-of-Flight Mass Spectrometry), although successfully used as a new sensitive screening technique for ACGs, also proved unsuitable for a definitive identification of this particular group [2]. These ACGs generally exhibit moderate inhibition potential of mitochondrial complex I [3,7,8], and thus have doubtful significant neurotoxicity [9]. However, as Annonaceae fruit pulps have been largely understudied so far (fruit pulps were addressed in only ~0.3% of peer-reviewed publications concerning ACGs!) [2], we pursued fractionation with a phytochemical and chemotaxonomic aim, focusing on these ACGs of weak polarity. 

## Results and Discussion

### Isolation and structural determination

A classical purification process of a CH_2_Cl_2_ extract of lyophilized pulp, using consecutive Sephadex LH-20^®^ and silica gel open column chromatographies followed by preparative C_18_ RP-HPLC, allowed isolation of three white waxy solids (**1+2**, **3+4**, **5+6**) from the apolar fractions. Each showed a single peak in analytical RP-HPLC, and reacted positively with Kedde reagent on thin layer chromatography. 

**Figure 1:**
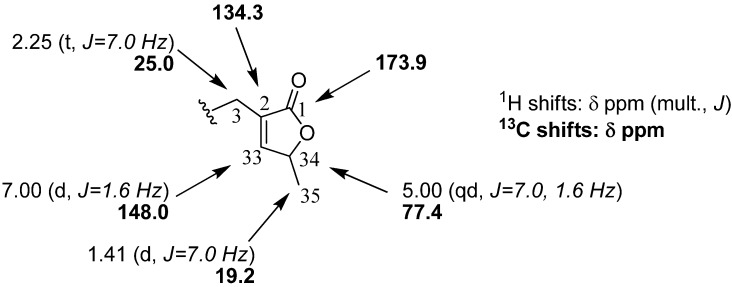
NMR shifts for the lactonic moiety of **1**+**2**.

Isolate **1+2**was obtained as a translucent waxy solid (10 mg). Molecular mass was determined as 530 by CI-MS ([M+H]^+^: *m/z* = 531) leading to the molecular formula C_35_H_62_O_3_. The existence of an α,β-unsaturated γ-lactone moiety, non hydroxylated at the C-4 position, was suggested by an IR carbonyl absorption at 1,750 cm^-1^ and UV λ_max _at 209.2 nm. It was confirmed by the ^1^H-NMR spectrum that showed seven protons at δ 7.00 (H-33, *d*), δ 5.00 (H-34, *qd*), δ 2.25 (2H, H-3, *t*) and δ 1.41 ppm (CH_3_-35, *d*) which were correlated on ^1^H-^13^C correlation spectra (HSQC, HMBC) with carbon atoms at δ 148.0 (C-33), 77.4 (C-34), 25.0 (C-3) and 19.2 ppm (CH_3_-35), along with signals at δ 134.3 (C-2) and 173.9 ppm (C-1) (Figure 1).

**Figure 2:**
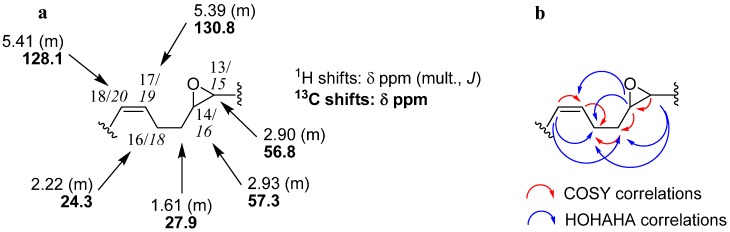
NMR shifts (**a**) and key ^1^H-^1^H correlations (**b**) for the epoxy-ene system of **1**+**2**.

The presence of an epoxy ring was suggested by ^1^H-NMR resonances at δ 2.90 and 2.93 ppm, each integrating for one proton (H-13/*H-15*, H-14/*H-16*), correlating with carbon atoms at δ 56.8 and 57.3 ppm (C-13/*C-15*, C-14/*C-16*). The presence of two multiplets at δ 5.39 and 5.41 ppm, each integrating for one vinylic proton (H-17/*H-19* and H-18/*H-20*, respectively) and correlating with signals at δ 128.1 (C-18/*C-20*) and δ 130.8 ppm (C-17/*C-19*), revealed the existence of a double bond on the aliphatic chain. The comparison of NMR data with that of other double-bond containing acetogenins of known configuration [10,11,12] revealed a *cis* configuration (carbon allylic resonance value at 27.3 ppm, characteristic of a *Z* relative configuration). The difference between the allylic methylenes’ ^13^C-NMR δ values suggested the proximity of the epoxy ring, which induces an upfield shift of the resonance of one of the two carbons (δ 24.3/27.9 ppm). The ^1^H-^1^H COSY (*J^1^*) and HOHAHA (*J^2^-J^3^*) spectra showed correlations between protons at δ 2.93 (H-14/*H-16*), δ 1.61 (H-15/*H-17*), δ 2.22 (H-16/*H-18*) and δ 5.39 ppm (H-17/*H-19*), suggesting that the double bond and the epoxy ring are separated by two methylene units (Figure 2).

**Figure 3:**
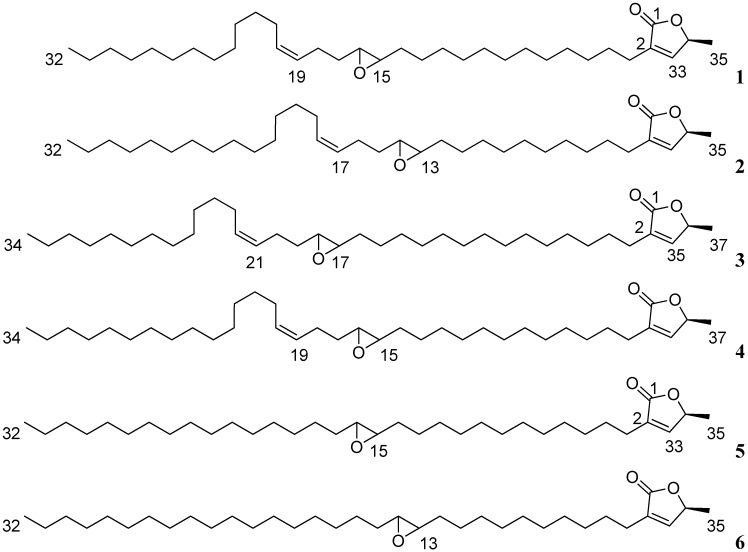
Obtained ACGs: Epomuricenins-A (**1**, epoxymurin-A) and -B (**2**); Epomusenins-A (**3**) and -B (**4**); Epomurinins-A (**5**) and -B (**6**).

Two series of characteristic, intense fragments on the EIMS spectrum [3], at *m/z* 321, 308, 307, 295 and at 293, 280, 279 and 267 suggested the existence of two isomers **1** and **2**, allowing localization of epoxy ring at *C-15/C-16* or at C-13/C-14, with the double bond location thus being *C-19/C-20* or C-17/C-18, respectively. These compounds were identified, by comparison with spectral data from the literature, as being epomuricenin-A (**1**, epoxymurin–A) and epomuricenin-B (**2**), respectively (Figure 3) [12,13,14]. The same mixture had been previously obtained in our laboratory, from the seeds of the species [10,12,13]. Confirmation of the identity of **1+2** was obtained using RP-HPLC, by co-injection with an authentic sample. Comparison of the optical rotation for **1+2** and the authentic sample suggested a *S* stereochemistry at C-34. Epomuricenin-A and -B were first isolated as a mixture in the seeds of *A. muricata* [10]. Epomuricenin-A was also obtained from the bark of the species, under the name epoxymurin-A, in a mixture with epoxymurin-B (epoxymurin-B bears an epoxide in positions C-17/C-18 and unsaturation in positions C-13/C-14) [14]. Characteristic EI-MS fragments of this last molecule were not observed, excluding its presence in the **1+2** mixture.

Isolate **3+4** (15 mg) was similarly identified as being a mixture, constituted of epomusenins-A (**3**) and -B (**4**) (Figure 3). Molecular mass was determined as 558 by CI-MS ([M+H]^+^: *m/z* = 559), corresponding to the molecular formula C_37_H_66_O_3_. 1D and 2D NMR data were quasi-identical to that of **1+2**, suggesting an ACG bearing an unsaturated butyrolactone, no hydroxyl group, an epoxide and an olefinic group separated by two carbon atoms. The EI-MS spectrum provided evidence for the existence of the two isomers, and allowed location of the epoxide and therefore of the double bond respectively at C-17/C-18 (*m/z* 336, 335, 323) and C-21/C-22 for epomusenin-A, and at C-15/C-16 (*m/z* 308, 307, 295) and C-19/C-20 for epomusenin-B. These molecules had been previously isolated in our laboratory as a mixture from the roots of the plant [13]. Confirmation of the identity of **3+4** was thus obtained by co-injection in RP-HPLC and by comparison of spectral data with that of the literature [13,15]. Optical rotation measurement suggested, as previously, a *S*-configuration for C-36. Epomusenins-A and -B were first described in the fruits of *Rollinia mucosa* [15].

**Figure 4:**
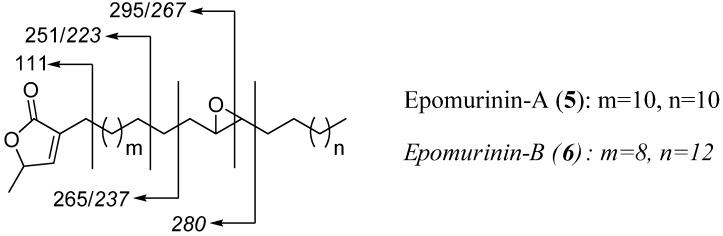
Key EI-MS fragmentation of ACGs (**5**+**6**).

For isolate **5**+**6** (2.5 mg), the CI-MS spectrum displayed a prominent peak at *m/z* = 533 (100%, [M+H]^+^) leading to the molecular formula C_35_H_64_O_3_. The low amount of material obtained unfortunately did not allow acquisition of a clear ^13^C-NMR spectrum. However, similar ^1^H-NMR data (Table 1) as above showed for this ACG the presence of an α,β-unsaturated γ-lactone moiety without a hydroxyl group at the C-4 position and of an epoxy ring (multiplet integrating for two protons at δ 2.92 ppm). No ^1^H-NMR signals for a double bond could be observed, as suggested by the 2 amu difference with epomuricenins-A and -B (**1**+**2**). EI-MS showed **5**+**6** to be a mixture, evidencing the presence of two compounds differing in the position of the epoxide group, at C-15/C-16 and at C-13/C14 for **5** and **6**, respectively (Figure 4). Compounds **5** and **6**, which we named epomurinins-A and-B are, to our knowledge, the first non-olefinic mono-epoxy ACGs described to date. They therefore are the first representatives of group 2b, in regard to the classification proposed by Bermejo *et al.* [4] in which group 2 (now proposed as group 2a) encloses mono-epoxy olefinic ACGs (type E-A) and group 3 comprises bis-epoxy ACGs (type E-B). In term of biogenetic relationships, no “naked” mono-ene ACG has been isolated to date. However, ACGs devoid of the THF moiety but bearing a *threo* vicinal diol, possibly arising from the opening of an isolated epoxy group, were described [4,12].

**Table 1:** ^1^H-NMR chemical shifts (δ ppm, *J* Hz) of ACGs **5**+**6**.

Position (5)	*Position (6)*	δ_H_, mult., ( *J*)	Position (5)	*Position (6)*	δ_H_, mult., ( *J*)
**3**	***3***	2.26, t (7.0)	**18 to 31**	***16 to 31***	1.25-1.19, m
**4**	***4***	1.55, m	**32**	***32***	0.87, t (6.6)
**5 to 13**	***5 to 11***	1.25-1.29, m	**33**	***33***	6.99, d (1.6)
**14**	***12***	1.32, m	**34**	***34***	5.01, dq (7.0; 1.6)
**15, 16**	***13, 14***	2.92, m			
**17**	***15***	1.61, m	**35**	***35***	1,41, d (7.0)

## Experimental

### General

Optical rotations were measured with a Schmidt-Haensch Polartronic I polarimeter. UV spectra were obtained in MeOH on a Philips PU 8720 spectrometer. IR spectra were recorded on a Bruker Vector 22 spectrometer. EI-MS spectra were recorded on an Automass multi (Thermo Finnigan, France), and CI-MS spectra with a Nermag-Sidar spectrometer. 1D and 2D NMR spectra were obtained with Bruker AC-200 (200 MHz) and Bruker AM-400 (400 MHz) spectrometers. TLC (thin layer chromatographies) were performed with Merck 60F254silica plates and revealed with the Kedde reagent (dinitrobenzoic acid 10% in MeOH, then KOH 2N in EtOH) for detection of unsaturated butyrolactones. For CC (column chromatography), Merck silica gels 60 (Merck 9385) and 60H (Merck 7736) and Sephadex^®^ LH-20 (Pharmacia) were used (VWR, Fontenay sous Bois, France). RP-HPLC was performed with a Waters SSV injector, a Waters 590 pump, a Prepak μ-Bondapak C_18_ column (25 × 100 mm), a UV Waters 484 detector (λ = 214 nm) and a ABB SE120 recorder (Waters Corporation, Milford, MA, USA and St.^.^ Quentin Falavier, France). Extraction solvents were purchased from Carlo-Erba (VWR) and HPLC grade MeOH from Prolabo. Water was purified by a Millipore water purification system and had a resistivity > 18 MΩ·cm^-1^. Authentic samples of ACGs where obtained by Dr C. Gleye [12,13] in the course of previous studies.

### Plant material

*A. muricata* L. fresh ripe fruits (soursop; 50 kg) were purchased on a market in Dakar (Senegal). Seeds and pericarps were carefully removed and pulp was lyophilized (final mass: 1.4 kg). 

### Extraction and isolation

Pulp was extracted with CH_2_Cl_2_ in a Soxhlet apparatus (10 L, 5 d). After solvent evaporation, an oily yellow residue, of strong and characteristic soursop odour, was obtained (14 g; yield: 1%; 280 mg/kg of fresh fruit). 13 g of this extract were chromatographed on silica gel 60 (AcOEt/iPrOH 9:1 Ò 1:9) to give eight fractions (F1 to F8). Fraction F1 (4.5 g) was subjected to CC on Sephadex^®^ LH-20 (CH_2_Cl_2_/MeOH 2:1), yielding four fractions (F1-1 to F1-4). F1-1 was subjected to CC on silica gel 60H (C_6_H_12_/AcOEt/iPrOH 13:0.3:0.3), yielding seven fractions (F1-1-1 to F1-1-7). Fractions F-1-1-4 (125 mg) and F-1-1-5 (103 mg) were merged and underwent CC on Sephadex^®^ LH-20 (CH_2_Cl_2_/MeOH 2:1), yielding four fractions (F-1-1-4/5-1 to F-1-1-4/5-4). F-1-1-4/5-4 (84 mg) was fractionated by RP-HPLC (MeOH/H_2_O 9:1, flow: 10 mL/min) to yield three single peak fractions of sufficient mass to allow spectroscopic analysis: **1**+**2** (10 mg, Rt = 22 min), **3**+**4** (15 mg, Rt = 34 min) and **5**+**6** (2.5 mg, Rt = 10 min).

### Spectral data

*Epomuricenins-A* and *-B* (**1**+**2**): White wax, [α]_D _= +8° (c 0.015, CHCl_3_); IR: 2,900, 2,830, 1,750, 1,460, 1,310, 1,200, 1,110, 1,060, 1,010 cm^-1^; UV (MeOH) λ_max_ (log ε): 209.2 (3.60); CI-MS (CH_4_): *m/z* = 531 [C_35_H_62_O_3_+H]^+^ (100%), 531, 513, 111; EI-MS (40 eV): *m/z* = 321, 293, 307, *308*, 295, *280*, *279*, *267*, 251, 237, *223*, 209, 195, 181, 167, 153, 139, 125, 111; ^1^H-NMR [CDCl_3_, 300 MHz, δ (ppm), *J* (Hz)]: δ 0.88 (3H, *t*, *J* = 7.0, H-32), 1.25-1.29 (36 H, *m*, H-5─H-11/*H-5─H-13*, H-21─H-31/*H-23─H-31*), 1.32 (2H, *m*, H-12/*H-14*), 1.41 (3H, *d*, *J* = 7.0, H-35), 1.50 (2H, *m*, H-20/*H-22*), 1.55 (2H, *m*, H-4), 1.61 (2H, *m*, H-15/*H-17*), 2.05 (2H, *m*, H-19/*H-21*), 2.22 (2H, *m*, H-16/*H-18*), 2.25 (2H, *t*, *J* = 7.0, H-3), 2.90 (1H, *m*, H-13/*H-15*), 2.93 (1H, *m*, H-14/*H-16*), 5.00 (1H, *qd*, *J* = 7.0 and 1.6, H-34), 5.39 (1H, *m*, H-17/*H-19*), 5.41 (1H, *m*, H-18/*H-20*), 7.00 (1H, *d*, *J* = 1.6, H-33). ^13^C-NMR (CDCl_3_, 200 MHz): 14.1 (C-32), 19.2 (C-35), 22.5 (C-31), 24.3 (C-16/*C-18*), 25.0 (C-3), 26.6-29.7 (C-22─C-29/*C-24─C-29*), 27.0 (C-4), 27.3 (C-19/*C-21*), 27.5-30.0 (C-5─C-12/*C-5─C-14*), 27.9 (C-15/*C-17*), 32.0 (C-30), 56.8 (C-13*/C-15*), 57.3 (C-14*/C-16*), 77.4 (C-34), 128.1 (C-18/*C-20*), 130.8 (C-17/*C-19*), 134.3 (C-2), 148.0 (C-33), 173.9 (C-1) (epomuricenin-B / *epomuricenin-A*) (*: interchangeable). 

*Epomusenins-A* and *-B* (**3**+**4**): White wax, [α]_D _= +9° (c 0.015, CHCl_3_); IR (film): 2,903, 2,835, 1,742, 1,463, 1,311, 1,213, 1,109, 1,050, 1,013 cm^-1^; UV (MeOH) λ_max_ (log ε): 208.6 (3.72); CI-MS (CH_4_): *m/z* = 559 [C_37_H_66_O_3_+H]^+^ (100%), 559, 541, 336, 312, 270, 130, 95; EI-MS (40 eV): *m/z* = 336, 323, 307, *308*, *295*, 279, 265, 251, 237, 223, 209, 195, 181, 167, 153, 139, 125, 111; ^1^H-NMR [CDCl_3_, 300 MHz, δ (ppm), *J* (Hz)]: δ 0.88 (3H, *t*, *J* = 6.7, H-34), 1.25-1.29 (40 H, *m*, H-5─H-13/*H-5─H-15*, H-23─H-33/*H-25─H-33*), 1.32 (2H, *m*, H-22/*H-24*), 1.50 (2H, *m*, H-14/*H-16*), 1.41 (3H, *d*, *J* = 7.0, H-37), 1.50 (2H, *m*, H-23/*H-25*), 1.55 (2H, *m*, H-4), 1.58 (2H, *m*, H-17/*H-19*), 2.05 (2H, *m*, H-21/*H-23*), 2.22 (2H, *m*, H-18/*H-20*), 2.25 (2H, *t*, J = 7.1, H-3), 2.91 (1H, *m*, H-15/*H-17*), 2.93 (1H, *m*, H-16/*H-18*), 5.00 (1H, *qd*, *J* = 6.8 and 1.5, H-36), 5.39 (1H, *m*, H-19/*H-21*), 5.41 (1H, *m*, H-20/*H-22*), 7.00 (1H, *d*, *J* = 1.5, H-35). ^13^C-NMR (CDCl_3_, 200 MHz): 14.1 (C-34), 19.2 (C-37), 22.5 (C-31), 24.3 (C-16/*C-18*), 25.2 (C-3), 26.6-29.7 (C-5─C-13/*C-5─C-15*, C-24─C-31/*C-26─C-31*), 27.4 (C-4, C-17/*C-19*, C-19/*C-21*), 27.7 (C-14/*C-16*), 32.0 (C-32), 56.7 (C-15*/C-17*), 57.0 (C-16*/C-18*), 77.4 (C-36), 128.0 (C-20/*C-22*), 131.0 (C-19/*C-21*), 134.2 (C-2), 148.0 (C-35), 173.9 (C-1) (epomusenin-B/ *epomusenin-A*) (*: interchangeable). Key COSY and HOHAHA correlations between protons at δ: 2.93, 1.61, 2.22, 5.41 ppm allowed relative positioning of epoxy and olefinic groups at a distance of two carbon atoms.

*Epomurinins-A* and *-B* (**5**+**6**): White wax, [α]_D _= +5° (c 0.0015, CHCl_3_); IR: 2,902, 2,835, 1,748, 1,460, 1,311, 1,212, 1,110, 1,050, 1,012 cm^-1^; UV (MeOH) λ_max_: 209.2; CI-MS (CH_4_): *m/z* = 533 [C_35_H_62_O_3_+H]^+^ (100%), 531, 513, 111; EI-MS (40 eV): *m/z* = 321, 293, 307, *308*, 295, *280*, *279*, *267*, 251, 237, *223*, 209, 195, 181, 167, 153, 139, 125, 111 (see Figure 2; epomuricenin-A/*epomuricenin-B*); ^1^H-NMR (CDCl_3_, 300 MHz): see Table 1. Comparison of [α]_D_ with that of **1**+**2**, **3**+**4** and of related compounds [12,13] suggests an *S* configuration for C-34. However, absolute configurations for C-13/C-14/*C-15/C-16* remain ambiguous; Mixtures of epimers for **5** and **6** can be suspected [16], this uncertainty remaining for the majority of epoxy-ACGs [4].

## Conclusion

**Figure 5:**
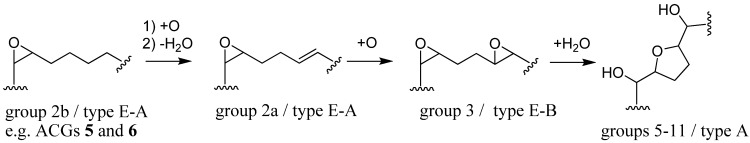
Putative pathway from group 2b to type A ACGs.

Isolation of type E Annonaceous acetogenins **1** to **6** offers a new insight into chemical composition of the fruit of *Annona muricata*, in relation to its probable role in the occurrence of sporadic atypical Parkinsonism in tropical areas. This result also suggests that fruit pulp might be a place for biosynthesis of ACGs and for their “maturation” into THF-bearing representatives such as annonacin. Epomurinins-A (**5**) and-B (**6**), the first “naked” mono-epoxy ACGs (proposed as group 2b ACGs), are minor compounds. Biogenesis of type-A (mono-THF) ACGs is proposed to result from oxygenation steps from dienic representatives [3,17]. In the hypothesis that ACGs **5** and **6** are not end-products, they might be precursors of several THF-bearing ACGs. To undergo such a fate, **5** and **6** would require further reduction (g group 2a – type E-A), epoxidation (g group 3 – type E-B) then hydratation steps, yielding type A ACGs (Figure 5) [3,17, *and see* 12]. Occurrence of these compounds would therefore illustrate the apparently poorly specific sequential distribution of biosynthetic steps [17,18] towards biologically efficient ACGs [3,4].

## Abbreviations

ACG: Annonaceous acetogenin; amu: atomic mass unit; CC: column chromatography; CI: chemical ionization; EI: electronic impact; MS: mass spectrometry; NMR: nuclear magnetic resonance; RP-HPLC: reversed-phase high performance liquid chromatography; TLC: thin layer chromatography.
